# Quinia—Its Therapeutical Action

**Published:** 1875-03

**Authors:** 


Quinia—Its Therapeutical Action.—We take from the *London Lancet* the following abstract of some lectures by M. See on the therapeutical action of quinia:

In health quinia has a three-fold action: 1, It diminishes the frequency and force of the heart's action; 2, It lowers the tension of the arterial system; and, 3, It lowers the temperature, or prevents its elevation by exercise. Its action on amæboid movements of white blood corpuscles is also recognized. Respecting the teachings of experience in the use of quinia in the varied forms of malarial fevers, he shows that the drug cannot be regarded as a specific, for it does not prevent the malarial poisoning when taken as a prophylactic; it does not prevent recurrence after a variable period, and it is useless in some of the most fatal forms, especially when the fever tends to assume a continued type.

Further, quinia has an equally beneficial effect in other fevers—periodic in character and beginning with a chill—as urethral fever from catheterism. In ague, he thinks that the effect of quinia is due to its three-fold action, exerted chiefly during the period of rigor; upon the heart it diminishes its frequency and force; on the peripheral arteries it lowers their tension and produces dilatation; on the spinal cord and vaso-motor centers, acting as a sedative, it tends to diminish their excitability; and lastly, it exerts a direct cooling action on the system generally.

In acute rheumatism M. See highly recommends quinia, and sees in its physiological action the most precise indications for its use.—*Detroit Review of Medicine and Pharmacy.*

